# Non-Invasive Evaluation of Hepatic Fibrosis: The Diagnostic Performance of Magnetic Resonance Elastography in Patients with Viral Hepatitis B or C

**DOI:** 10.1371/journal.pone.0140068

**Published:** 2015-10-15

**Authors:** Wen-Pei Wu, Chen-Te Chou, Ran-Chou Chen, Chih-Wei Lee, Kwo-Whei Lee, Hwa-Koon Wu

**Affiliations:** 1 Department of Diagnostic Radiology, Lu-Kang Christian Hospital, Changhua City, Taiwan, R.O.C; 2 Department of Biomedical Imaging and Radiological Sciences, National Yang-Ming University, Taipei City, Taiwan, R.O.C; 3 Department of Radiology, Chang-Hua Christian Hospital, Changhua City, Taiwan, R.O.C; 4 Department of Radiology, Taipei City Hospital, Taipei City, Taiwan, R.O.C; National Taiwan University Hospital, TAIWAN

## Abstract

**Purpose:**

To compare the accuracy of magnetic resonance elastography (MRE) with that of aspartate aminotransferase-to-platelet ratio index (APRI) for estimating the stage of hepatic fibrosis in patients with chronic hepatitis B virus (HBV) or chronic hepatitis C virus (HCV) infection.

**Materials and Methods:**

We retrospectively enrolled 160 patients with chronic hepatitis and 25 healthy living liver donors. Fibrosis stage (METAVIR, F0 to F4) was determined histopathologically for all patients. APRI was recorded at the time of histopathologic examination and liver stiffness values were measured on MRE quantitative stiffness maps. The cutoff values, sensitivity, and specificity of MRE and APRI for each fibrosis stage were determined using receiver operating characteristic (ROC) analysis.

**Results:**

MRE had a significantly greater area under the ROC curve than APRI score for discriminating among METAVIR stages F2-F4. Using a cutoff value of 2.80 kPa, MRE had a sensitivity of 94.4% and a specificity of 97.8% for detecting significant fibrosis (≥F2). There were no significant differences in fibrosis stage between patients with HBV and those with HCV infection. For ≥F2, the cutoffs were 2.47 kPa (100% sensitivity), 2.80 kP (maximum sum of sensitivity and specificity), and 3.70 kPa (100% specificity).

**Conclusions:**

MRE is a more accurate modality than APRI for detecting significant fibrosis in patients with chronic HBV or HCV infection. Antiviral treatment should be considered in patients with liver stiffness values ≥ 2.8 kPa.

## Introduction

Viral hepatitis places a heavy burden on health care systems because of the high costs of treatment of liver cancer and liver cirrhosis. Approximately five hundred million people worldwide are chronically infected with viral hepatitis B (HBV) or viral hepatitis C (HCV). Identification of significant liver fibrosis in patients with HBV or HCV infection is crucial to establish the timing of antiviral treatment.[1, 2]

Liver biopsy is the gold standard for determining fibrosis stage. However, it is an invasive procedure and has several limitations, including a high inter-observer variability and significant sampling errors of up to 14.5%-25%.[3, 4] Non-invasive alternatives to liver biopsy include radiological examinations and the use of biochemical scores, such as the aspartate aminotransferase—to-platelet ratio index (APRI). A number of studies in western countries have shown that among all currently used noninvasive methods magnetic resonance elastography (MRE) has the highest correlation with liver fibrosis stage in patients with chronic HCV infection.[5–10] However, in Asia HBV rather than HCV infection is the leading cause of chronic hepatitis, cirrhosis and hepatocellular carcinoma [11]. Microscopically, chronic HCV infection is characterized by the triad of lymphocyte nodular inflammation in portal tracts, the presence of steatosis and bile duct damage whereas the presence of “ground-glass” hepatocytes is the histologic hallmark of chronic hepatitis B infection.[12–14] Liver stiffness values as measured by transient elastography (TS) or acoustic radiation force impulse (ARFI) imaging can also differ between patients with HBV infection and those with infection due to HCV, making it difficult to differentiate between different stages of fibrosis in these two groups of patients.[15–17]

Magnetic resonance elastography (MRE) has been shown to be a more reliable method than TS or ARFI imaging for measuring liver stiffness in patients with chronic hepatitis. Nonetheless, the effect of several clinical, biological and histopathological factors such as necroinflammatory activity, liver steatosis and biochemical profiles on liver stiffness measurements have not been comprehensively considered when evaluating the diagnostic accuracy of MRE. The most common finding in patients with chronic HBV and HCV infection is steatosis. Some studies have shown that the accuracy of liver stiffness measurement obtained by ultrasound elastography is influenced by the presence of liver steatosis.[18–20] Therefore, understanding whether the presence of steatosis affects the accuracy of MRE in measuring liver stiffness and hence determining the stage of fibrosis of the liver is of particular importance.

The purpose of this study was to compare the accuracy of MRE with that of APRI for estimating the stage of hepatic fibrosis in patients with chronic HBV or HCV infection. The results of histopathologic analysis were used as the reference standard and the optimal cutoff values of liver stiffness for different stages of liver fibrosis were defined. We also investigated whether the presence of hepatic steatosis affected the accuracy of MRE measurements.

## Materials and Methods

### Patients

**Fig 1:**
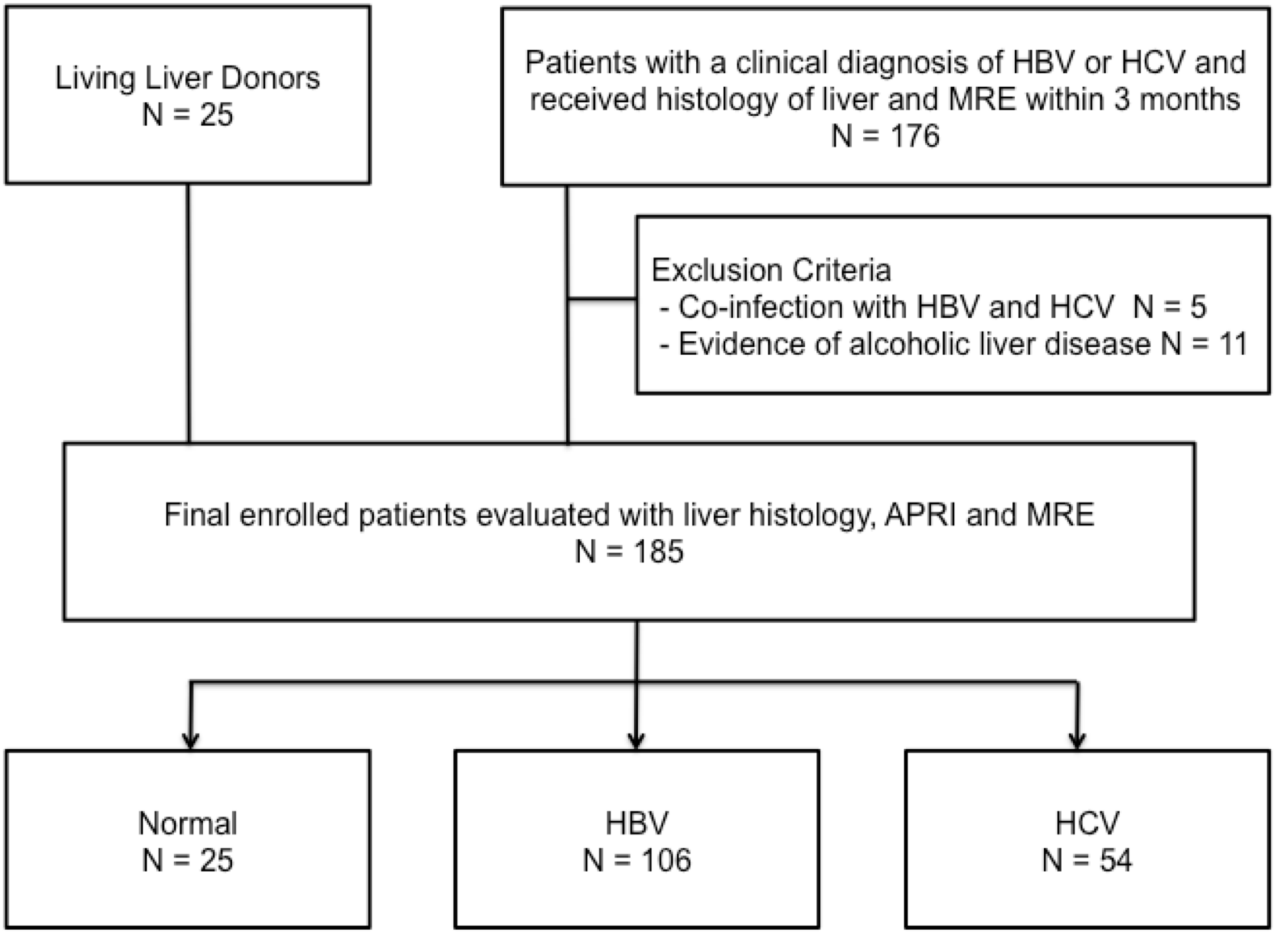
Flow diagram of patient selection. HBV = chronic hepatitis B; HCV = chronic hepatitis C; APRI = aspartate aminotransferase to the platelet ratio index; MRE = MR elastography.

This retrospective study was approved by the institutional review board of Changhua Christian Hospital. The need for written informed consent was waived by the committee. All patients with chronic viral hepatitis (HBV or HCV) infection who underwent histopathological examination during the period January 2011 to July 2013 and who underwent MRE within 3 months of the histopathological examination were eligible for enrolment in this study. (Fig 1) HBV was defined in patients who tested positive for hepatitis B surface antigen and HCV was defined in patients who tested positive for both anti-HCV and HCV-RNA. Exclusion criteria included evidence of alcoholic liver disease and co-infection with hepatitis B and hepatitis C. Age, gender, height, weight, body mass index (BMI), serum aspartate aminotransferase (AST), alanine aminotransferase (ALT), total bilirubin and platelet counts were recorded, and APRI scores were calculated at the time of histopathological examination.

A total of 160 patients with viral hepatitis (121 men and 39 women; mean age, 59.1 years; age range 26–80 years) fulfilled the eligibility requirements and were enrolled in the study (Fig 1). Of the 160 patients, 106 had chronic HBV infection and 54 had chronic HCV infection. We also enrolled 25 living liver donors (13 men and 12 women; mean age, 29 years; 21–45 years) to serve as the control group.

### MRI

All examinations were performed on a 1.5T MR system (Avanto, Siemens, Erlangen, Germany) for acquisition of routine clinical MR and MRE images. A 16-channel phased-array body coil was used for signal reception.

MRE was performed using an acoustic driver system (Rochester, MN, USA). During the acquisition, a 19-cm-diameter, 1.5-cm-thick cylindrical passive driver was placed against the right chest wall over the liver. Continuous acoustic vibration at 60 Hz transmitted from an active driver was used to produce propagating shear waves in the liver. The propagating shear waves were imaged with axial 2D gradient-echo sequencing. The parameters of the MRE sequence were as follows: TR/TE, 50/22.7; flip angle, 25°; bandwidth, 260 Hz/pixel; imaging frequency, 63.5MHz; acquisition matrix, 256 × 64; section thickness, 7 mm; and FOV, 400 × 400 mm^2^. The scanning time of each axial slice was 21 seconds per breath-hold. A total of five axial slices were acquired. All post-processing steps were applied automatically and displayed liver stiffness in kilopascals. A confidence map providing regions with adequate wave amplitude was generated automatically by the MRE software.

### MRE Analyses

**Fig 2:**
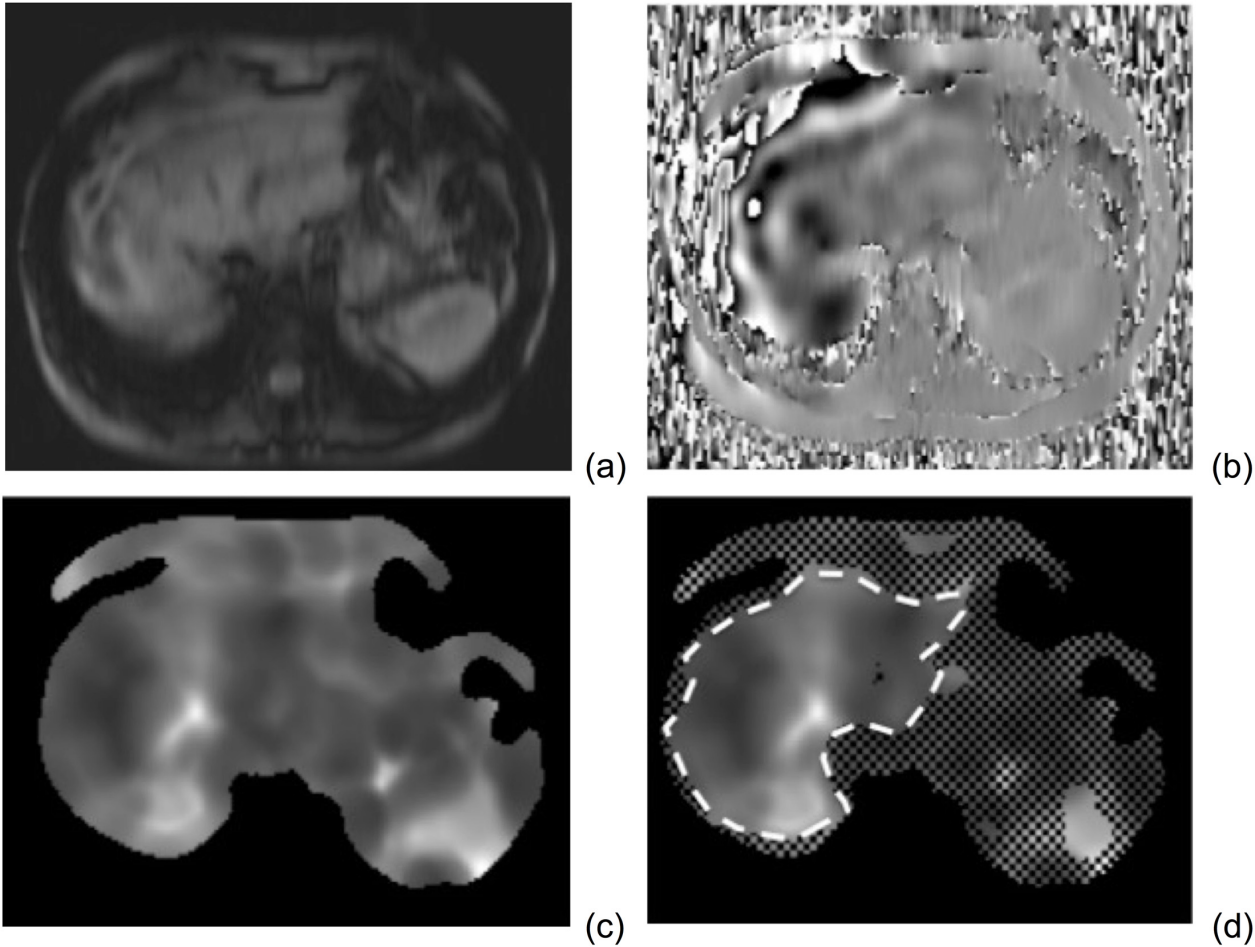
MRE data in a 46-year-old female with hepatitis B. (a) magnitude image,.(b) wave image, (c) stiffness map, and (d) confidence map. All images are at the same level. The dotted lines on the confidence maps represent the liver outlines of the corresponding patient’s MR images. The overall mean stiffness value on a total of 5 MRE images was 2.34 kPa. Liver biopsy confirmed fibrosis stage F1.

All analyses were performed on a dual-screen diagnostic workstation (GE Healthcare, Milwaukee, WI, USA). One attending abdominal radiologist, who specializes in liver MRI, performed the quantitative analyses of MRE images. The radiologist was blinded to the patients' clinical data and histopathological results. For the measurement of liver stiffness, the wave images were first checked for adequate wave quality. On each axial image of the confidence map, the ROI (region of interest) was manually drawn to include only the parenchyma of the liver (Fig 2) The areas on the confidence map that were determined to be invalid were excluded. The mean stiffness value (in kilopascal) for each elastographic image (five slices per patient) was recorded. Care was also taken to avoid artifacts such as wave interference, the edges of the liver, large blood vessels and hepatic tumors. The mean stiffness value (in kilopascals) for each elastographic image (five slices per patient) was recorded. The overall mean stiffness value of the liver parenchyma was calculated by averaging the mean stiffness values on the 5 slices in each patient.

### APRI Analysis

The upper limits of normal (ULN) aspartate aminotransferase (AST, in international units per liter) are 38 U/L for men and 32 U/L for women. The APRI was determined as follows: [(AST/ ULN)/platelet count (10^9^/L)] x 100. [21]

### Histopathological Analysis

The diagnosis of hepatic fibrosis was based on the results of histopathological analysis of percutaneous liver biopsy specimens (n = 74), surgical resection specimens (n = 81), liver tissue samples from living donors (n = 25) or samples from transplanted liver (n = 5). Tissue samples were fixed in buffered formalin and embedded in paraffin. Samples were subjected to standard techniques for Hematoxylin and eosin (H&E) and Masson trichrome staining and slides were reviewed by an experienced attending pathologist who was blinded to the patients’ clinical information and MR imaging results. All liver samples were considered adequate based on specimen size (≥10mm) and number of portal tracts (≥5). Liver fibrosis was staged according to the METAVIR scoring system, which ranges from F0 to F4 (F0 = no fibrosis; F1 = portal fibrosis without septa; F2 = portal fibrosis with a few septa; F3 = numerous septa without cirrhosis; F4 = cirrhosis). A METAVIR fibrosis stage of F2 or higher was considered to represent significant fibrosis. [22] Necroinflammatory activity was graded as follows: A0, none; A1, mild activity; A2, moderate activity; and A3, severe activity. Liver steatosis was graded based on the percentage of hepatocytes with macrovesicular fat: grade 0 (none or less than 5%); grade 1 (mild steatosis, 6% to 33%); grade 2 (moderate steatosis, 34% to 66%); and grade 3 (severe steatosis, greater than 66%).

### Statistical Analysis

Data are expressed as mean ± standard deviation, 95% confidence interval (CI) or number (n), as appropriate. Box plots were used to compare liver stiffness values measured on MRE images and APRI values with METAVIR scores. The relationships between MRE and fibrosis stage and APRI and fibrosis stage were assessed using Spearman's correlation coefficient. Sensitivity, specificity, positive and negative predictive values and positive and negative likelihood ratios of MRE and APRI for estimating fibrosis stage were evaluated by calculating the areas under the receiver operating characteristic (ROC) curves (Az). The optimal cutoff values were chosen by maximizing the Youden index on the estimated curves (sensitivity + specificity-1). Concordance was defined as the rate of patients who were classified correctly (true positive and true negative). Az values of MRE and APRI were also compared using MedCalc software (version 13.1.2; MedCalc, Mariakerke, Belgium). Different cutoff values for liver stiffness were chosen to obtain 100% sensitivity, 100% specificity, and a maximum sum of sensitivity and specificity. Patient variables were first assessed with MRE-measured liver stiffness values using univariate linear regression analysis. Significant variables in the univariate analysis were then included in a multivariate linear regression model to identify factors independently associated with liver stiffness. The statistical significance of liver stiffness differences between patients with or without steatosis at each stage of liver fibrosis was evaluated by means of the Mann-Whitney U test. A two-tailed *p* value less than 0.05 was considered statistically significant. With the exception of MedCalc software to compare Az values of MRE and APRI, all statistical analyses were performed with the statistical software package SPSS for Windows (version 20.0, Chicago, IL).

## Results

### Baseline characteristics of patients

The clinical and biochemical characteristics of the patients in this study are summarized in Table 1. The distribution of fibrosis stage among the 185 patients is as follows: F0, n = 25 (13.5%); F1, n = 18 (9.7%); F2, n = 39 (21.1%); F3, n = 39 (21.1%); and F4, n = 64 (34.6%). Liver steatosis was present in 89 patients (48.1%).

**Table 1:** Patient characteristics of the study population with different stages of liver fibrosis.

Fibrosis stage	F0	F1	F2	F3	F4
**Number of Patients**	25	18	39	39	64
**Age (years)**	32.7 ± 13.0	57.6 ± 9.7	56.1 ± 13.5	57.7 ± 12.0	61.8 ± 9.4
**Gender**					
** Male**	13	14	32	31	45
** Female**	12	4	7	8	19
**Body mass index (kg/m** ^**2**^ **)**	23.4 ± 3.5	25.3 ± 4.6	23.1 ± 3.2	24.2± 3.7	24.0 ± 3.7
**Etiology**					
** HBV**	-	13	29	26	38
** HCV**	-	5	10	13	26
**AST (xULN)** ^a^	0.70 ± 0.47	1.19 ± 0.75	1.61 ± 1.35	1.72 ± 1.21	1.71 ± 1.56
**ALT (xULN)** ^b^	0.75 ± 0.64	1.12 ± 0.94	1.36 ± 1.41	1.42 ± 1.13	1.48 ± 1.39
**Bilirubin (mg/dL)**	0.65 ± 0.26	0.91 ± 0.44	1.02 ± 1.04	0.92 ± 0.36	1.00 ± 0.49
**Platelet count (x10** ^**9**^ **/L)**	250.1 ± 60.9	183.8 ± 68.1	152.2 ± 50.3	133.3± 65.7	114.8 ± 43.0
**Steatosis (grade 0–3)**					
** Grade 0 (none)**	22 (79%)	11 (61%)	14 (36%)	18 (46%)	31 (50.8%)
** Grade 1 (mild)**	6(21%)	7 (39%)	20 (51%)	16 (41%)	23 (37.7%)
** Grade 2 (moderate)**	-	-	3 (8%)	2 (5%)	4 (6.6%)
** Grade 3 (severe)**	-	-	2 (5%)	3 (8%)	3 (4.9%)

Ages, body mass index values, AST, ALT, bilirubin levels and platelet counts are presented as mean ± 1 Standard Deviation.

ALT, serum alanine aminotransferase; AST, serum aspartate aminotransferase;

^a^AST reference value: 38 U/l (men), 32 U/l(women)

^b^ALT reference values: 45 U/l (men), 40 U/l(women); ULN: upper limit of normal

### Correlation between Magnetic Resonance Elastography, APRI and fibrosis stage

**Fig 3:**
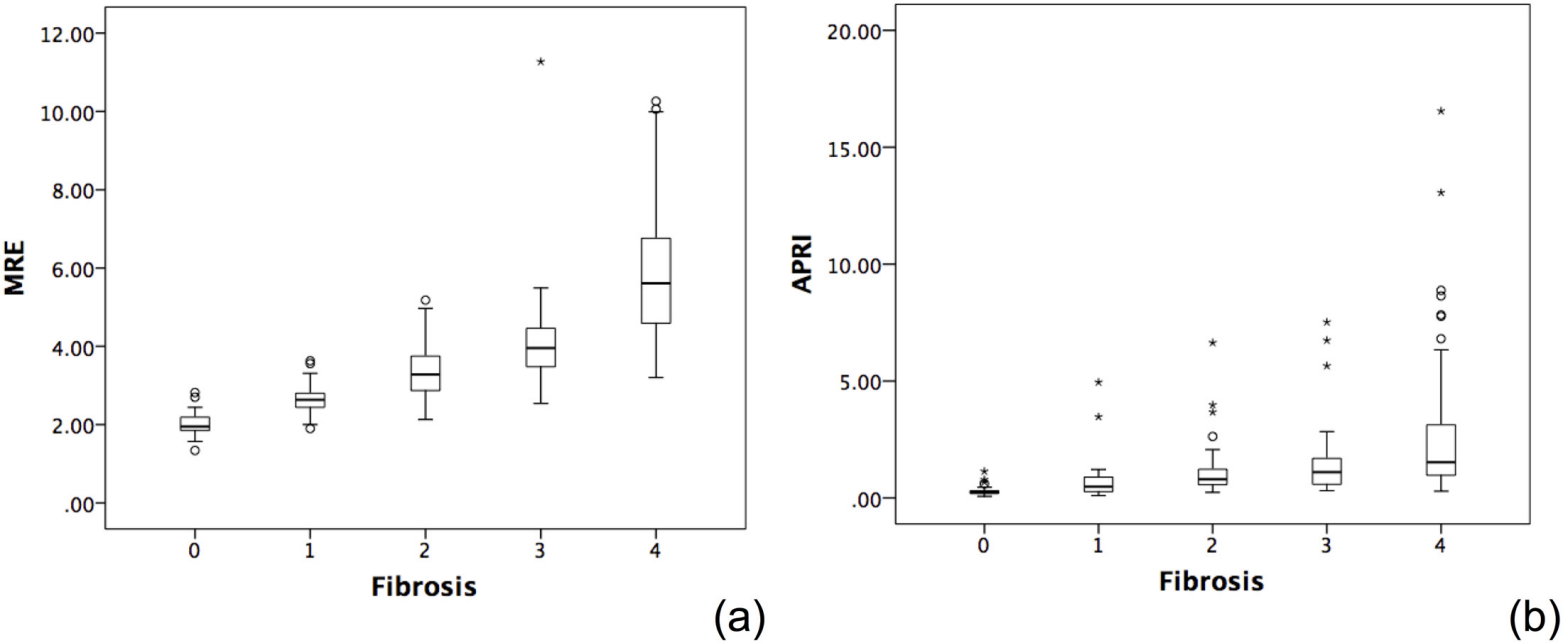
Box plots. (a) liver stiffness values measured by MRE and (b) APRI values according to each METAVIR fibrosis stage (F0 to F4). The mean liver stiffness scores and APRI values increased with increasing METAVIR stage of fibrosis. (Spearman’s risk correlation coefficient = 0.85 and 0.42, respectively) The horizontal line through each box represents the median and each box represents data from the 25^th^ to the 75^th^ percentile. The separate asterisks and circles represent outliers.

Liver stiffness values in the study population ranged from 1.34 kPa to 10.26 kPa (mean, 3.9±1.68kPa; 95% CI, 0.61–7.19kPa) and increased with increasing liver fibrosis stage (Fig 3a). There was excellent correlation between MRE liver stiffness values and liver fibrosis stage (Spearman’s rank correlation coefficient, ρ, = 0.850, p<0.001). MRE-measured liver stiffness values differed significantly between all stages of liver fibrosis (p<0.001).

The APRI scores ranged from 0.07 to 16.55 (mean, 1.47±1.95; 95% CI, 0–3.34) and increased with increasing liver fibrosis stage. (Fig 3b) A substantial positive correlation between APRI score and stage of fibrosis was also observed. (ρ = 0.42, *p*<0.001). APRI scores differed significantly between all stages of fibrosis with the exception of F2 and F3 (p = 0.274).

### Diagnostic accuracy of MRE and APRI for estimating fibrosis stage in patients with HBV and HCV

The diagnostic accuracy of both modalities in estimating each fibrosis stage in patients with HBV and HCV is summarized in Table 2. MRE had a significantly greater area under the ROC curve than APRI score for discriminating among METAVIR stages F2-F4.

**Table 2:** ROC analysis to determine the accuracy of MRE and APRI in predicting METAVIR fibrosis stage in patients with HBV or HCV.

Category	Fibrosis	Az	Cutoff (kPa)	Sn (%)	Sp (%)	PPV (%)	NPV (%)	+LR	-LR	Concordance (%)	*P* ^a^
**All patients**											
** MRE**	≥F1	0.993	2.46	95.1	100	100	82.4	∞	0.04	96.8	0.123
	≥F2	0.984	2.80	94.4	97.8	98.5	83.3	19.83	0.06	96.8	0.0001
	≥F3	0.949	3.77	82.9	91.8	92.3	82.9	10.20	0.17	87.6	<0.0001
	F4	0.962	4.09	90.9	86.3	78.4	94.6	6.85	0.11	88.1	<0.0001
** APRI**	≥F1	0.877	0.465	88.9	96.4	99.3	60.0	24.79	0.11	89.7	
	≥F2	0.774	0.985	62.5	85.7	92.7	43.8	4.21	0.42	69.2	
	≥F3	0.708	1.08	63.8	73.3	75.0	63.9	2.40	0.47	69.2	
	F4	0.801	1.09	69.7	62.6	51.2	80.8	0.03	0.46	67.0	
**Patients with HBV**											***P*** ^b^
** MRE**	≥F1	0.994	2.48	95.2	100	100	83.9	∞	0.05	96.2	0.996
	≥F2	0.980	2.73	95.7	94.9	97.8	90.2	18.65	0.05	95.4	0.991
	≥F3	0.942	3.76	81.3	94.0	92.9	84	13.61	0.20	87.8	0.973
	F4	0.960	4.16	91.9	89.4	77.3	96.6	7.62	0.09	90.1	0.984
**Patients with HCV**											
** MRE**	≥F1	0.996	2.47	98.1	100	100	96.4	∞	0.02	98.7	
	≥F2	0.986	2.73	95.7	90.6	93.8	93.6	10.21	0.05	93.7	
	≥F3	0.976	3.71	91.7	90.7	89.2	92.9	9.85	0.09	91.1	
	F4	0.975	3.83	100	88.5	81.8	100	8.67	∞	92.4	

MRE = MR Elastography; Az = area under the curve; kPa = kilopascal; Sn, sensitivity; Sp, specificity; PPV, positive predictive value; NPV, negative predictive value; +LR, positive likelihood ratio; -LR, negative likelihood ratio;

Concordance represents the rate of patients who were classified correctly (true positive + true negative).

^a^ Represents the comparison of the diagnostic performance of MRE and APRI in all patients for each corresponding fibrosis stage.

^b^ Represents the comparison of the diagnostic performance of MRE between patients with HBV and HCV for each corresponding fibrosis stage.

Using a cutoff value of 2.80 kPa, MRE had a sensitivity of 94.4%, a specificity of 97.8%, a PPV of 98.5% and a concordance rate of 96.8% for detecting significant fibrosis (≥F2). In contrast, with a cut off value of 0.985, APRI had a sensitivity of 62.5%, a specificity of 85.7%, a PPV of 93.8%, a concordance rate of 69.2% and an Az of 0.774±0.052. With a cut off value of 4.09 kPa, MRE had a sensitivity of 90.9%, a specificity of 96.3%, a PPV of 51.2%, a concordance rate of 67.0% and an Az of 0.962±0.011 in predicting liver cirrhosis (F4).

Mean liver stiffness values for patients with a METAVIR score of F1 were 2.47±0.24 kPa for patients with HBV and 2.72±0.234 kPa for patients with HCV (p = 0.169). For patients with a score of F2, the mean stiffness values were 3.25±0.61 kPa for patients with HBV and 3.43±0.58 kPa for patients with HCV (p = 0.182). For patients with a METAVIR score of F3, the mean stiffness values were 3.92±0.70 kPa for HBV patients and 3.97±0.54 kPa for HCV patients (p = 0.89). And for patients with a score of F4, the mean values were 4.21±1.52 kPa for patients with HBV and 4.54±1.75 for patients with HCV (p = 0.53). There were no significant differences in liver stiffness for each METAVIR stage between patients with hepatitis B and those with hepatitis C.

The diagnostic performance of MRE in patients with HBV was similar to that in patients with HCV. There were no significant differences in Az for the diagnosis of each METAVIR fibrosis stage between patients with HBV and those with HCV. The optimal cutoff values to classify each METAVIR fibrosis stage were similar between patients with HBV and those with HCV with the exception of F4, for which the optimal cutoff value was 4.16 kPa for patients with HBV and 3.83 kPa for patients with HCV.

### Optimal liver stiffness cutoff values measured by MR Elastography

Based on the ROC curves for each METAVIR fibrosis stages, three optimal liver stiffness cutoff values were defined: cutoff values with 100% sensitivity, cutoff values that maximize the sum of sensitivity and specificity, cutoff values with 100% specificity (Table 3). The optimal cutoff values were 1.99, 2.46 and 2.46 kPa for fibrosis stages ≧F1; 2.47, 2.80 and 3.70 kPa for significant fibrosis (≧F2); and 3.15, 4.09 and 5.50 kPa for cirrhosis (F4).

**Table 3:** Optimal liver stiffness cutoff values measured by MR Elastography for different degrees of liver fibrosis.

Fibrosis	Category*	Cutoff (kPa)	Sn	Sp	PPV	NPV	+LR	-LR	Concordance
**≥F1**	Sn	1.99	100%	60.7%	93.5%	100%	2.55	Infinite	94.1%
	Sn+Sp	2.46	95.1%	100%	100%	82.4%	Infinite	0.04	96.8%
	Sp	2.46	95.1%	100%	100%	82.4%	Infinite	0.04	96.8%
**≥F2**	Sn	2.47	100%	75.6%	92.7%	100%	4.09	Infinite	94.1%
	Sn+Sp	2.80	94.4%	97.8%	98.5%	83.3%	21.86	0.03	96.8%
	Sp	3.70	67.9%	100%	100%	50.0%	Infinite	0.32	75.7%
**≥F3**	Sn	2.54	100%	43.5%	67.6%	100%	1.77	Infinite	74.1%
	Sn+Sp	3.77	82.9%	91.8%	92.3%	82.9%	10.20	0.17	87.6%
	Sp	5.39	34.0%	100%	100%	56.3%	Infinite	0.66	64.3%
**F4**	Sn	3.15	100%	55.4%	54.2%	100%	2.24	Infinite	70.8%
	Sn+Sp	4.09	90.9%	86.3%	78.4%	94.6%	6.85	0.11	88.1%
	Sp	5.50	50.0%	100%	100%	79.1%	Infinite	0.50	82.7%

kPa, kilopascal; Sn, sensitivity; Sp, specificity; PPV, positive predictive value; NPV, negative predictive value; LR (+), positive likelihood ratio; LR (-), negative likelihood ratio.

Concordance represents the rate of patients who were classified correctly (true positive + true negative).

*Sn, 100% sensitivity; Sn+Sp, a maximum sum of sensitivity and specificity; Sp, 100% specificity.

### Effects of clinical, biological and histopathological characteristics on liver stiffness

Univariate and subsequent multivariate analyses of clinical, biological, and histopathological characteristics of patients were performed to identify independent variables associated with liver stiffness as measured by MRE. The results are shown in Table 4. Liver fibrosis (p<0.0001), necroinflammatory activity (p<0.0001), ALT (p = 0.04) and platelet count (p = 0.03) were found to be important independent confounding factors of liver stiffness measurements using MRE.

**Table 4:** Influences of clinical, biological and histolopathological parameters on liver stiffness measurement using MR Elastography.

Variables	Univariate	*P* value	Multivariate	*P* value
Age (years)	0.052(0.037–0.066)	<0.0001	0.008(0.004–0.020)	0.194
Male	0.275(-0.259–0.808)	0.311	-	-
BMI	0.019(-0.052–0.090)	0.604	-	-
Etiology (HBV vs. HCV)	0.342(-0.174–0.858)	0.193	-	-
Fibrosis (F1-F4)	0.918(0.814–1.021)	<0.0001	0.738(0.589–0.887)	<0.0001
Activity (A0-A3)	1.201(0.961–1.442)	<0.0001	0.437(0.213–0.661)	<0.0001
Steatosis (S0-S3)	0.228(-0.085–0.540)	0.153	-	-
Iron (n vs. y)	0.973(-0.042–1.987)	0.060	-	-
AST (xULN)	0.434(0.260–0.608)	<0.001	0.165(-0.051–0.382)	0.113
ALT (xULN)	0.333(0.147–0.518)	0.001	0.227(0.010–0.444)	0.040
Bilirubin	0.553(0.160–0.945)	0.006	0.121(-0.120–0.361)	0.324
Platelet	-0.013(-0.016–0.011)	<0.0001	-0.003(-0.006–0.000)	0.030

The results of the univariate and multivariate linear analyses are expressed as unstandardized B coefficients with 95% confidence intervals.

### Comparisons of liver stiffness between patients with and without liver steatosis

Liver stiffness values increased with increasing fibrosis stage in patients with and in those without liver steatosis (Table 5). In the subgroup of patients with METAVIR stage F0-F1, MRE-measured liver stiffness values were marginally higher in patients with liver steatosis than in patients without liver steatosis (*p* = 0.048). However, in the subgroups of patients with F2-F3 and F4, there were no significant differences in liver stiffness values between patients with and those without liver steatosis (*p* = 0.641 and 0.079). Using ROC curve analysis, we found that the optimal cut off value for discriminating significant fibrosis (≥F2) in patients with steatosis was 2.83 kPa.

**Table 5:** Analysis of the effect of liver steatosis on liver stiffness estimated by MR Elastogrpahy for each fibrosis stage.

	Liver stiffness		
	Liver steatosis (n = 89)	None (n = 96)	*p* value
**F0-F1** (kPa)	2.42±0.42	2.20±0.47	0.048
**F2-F3** (kPa)	3.73±1.30	3.69±0.68	0.641
**F4** (kPa)	5.62±1.79	6.06±1.47	0.079

Values are depicted as mean ± standard deviation. kPa = kilopascal.

## Discussion

In this study, we evaluated and compared the diagnostic accuracy of MRE and APRI in estimating liver fibrosis in patients with chronic HBV or HCV infection. We found that both MRE and APRI showed a positive correlation with histopathologically determined METAVIR fibrosis stage, but that APRI score was less accurate than MRE in estimating the degree of fibrosis. Our results are in agreement with those presented in previous studies.[7, 23–26] Based on the ROC curve analysis, MRE is more accurate than APRI in differentiating significant fibrosis (≥F2) from normal/mild fibrosis (F0/1). Using a cutoff value of 2.80kPa, MRE had a sensitivity of 94.4% and a specificity of 97.8% for differentiating significant fibrosis from normal liver/mild fibrosis. This high rate of accuracy is important as treatment with antiviral medication is indicated only in patients for whom liver biopsy specimens indicate evidence of significant fibrosis, according to guidelines published by the American Association for the Study of Liver Diseases (AASLD) and the European Association for the Study of the Liver (EASL). [22] Long-term antiviral therapy can result in a regression, but not reversal of cirrhosis in chronic hepatitis B patients.[27, 28] Therefore, regular follow-up is mandatory for patients with chronic viral hepatitis. Our results indicate that MRE is a reliable noninvasive method for long-term monitoring of hepatic fibrosis condition in these patients.

In our study, we found that liver stiffness and necroinflammatory activity were the strongest independent confounding factors associated with MRE-measured liver stiffness. Previous studies have also suggested that necroinflammatory activity resulting from viral hepatitis also influences transient elastography-measured liver stiffness and should be regarded as a strong confounding factor.[29] Ichikawa et al. reported that necroinflammatory activity in patients with hepatitis independently affected MRE-measured liver stiffness values.[30] However, a recent meta-analysis revealed that the diagnostic accuracy of MRE for the detection of significant fibrosis was not influenced significantly by the presence of severe necroinflammation.[31] Prospective studies with large patient populations may be needed to further evaluate the effect of necroinflammation on measurement of liver stiffness.

Some studies on the value of liver stiffness measurements obtained by transient elastography in patients with chronic hepatitis due to HBV or HCV have reported conflicting results. [5, 15–17, 32] In our study, we compared liver stiffness values between patients with HBV and those with HCV infection and found that there were no significant differences in stiffness values between the two groups of patients with different METAVIR fibrosis stages. Our results are in agreement with those reported in previous studies using transient elastography (TE) and acoustic radiation force impulse (ARFI) elastography. [5, 15, 16, 32] However, in our study, the optimal cutoff values for liver cirrhosis were similar between patients with HBV and those with HCV infection with the exception of F4, for which the cutoff value was 4.16 kPa for patients with HBV and 3.83kPa for patients with HCV. Further studies comprising a larger patient population are needed to better understand this result.

The influence of liver steatosis on liver stiffness during MRE measurement was also evaluated in this study. Our results show that, among patients with a histopathologically determined fibrosis stage of F0-F1, liver stiffness values were marginally higher in patients with liver steatosis than in those without steatosis (p = 0.048). No significant differences in liver stiffness were noted between steatotic and non-steatotic patients in terms of fibrosis stages F2-F4. Interestingly, the cut off value of liver stiffness measured by MRE to discriminate significant liver fibrosis was very similar between patients with liver steatosis (2.83 kPa) and patients with chronic hepatitis (2.80 kPa). According to our results, liver steatosis has a negligible effect on the cut off value to discriminate significant liver fibrosis.

**Fig 4:**
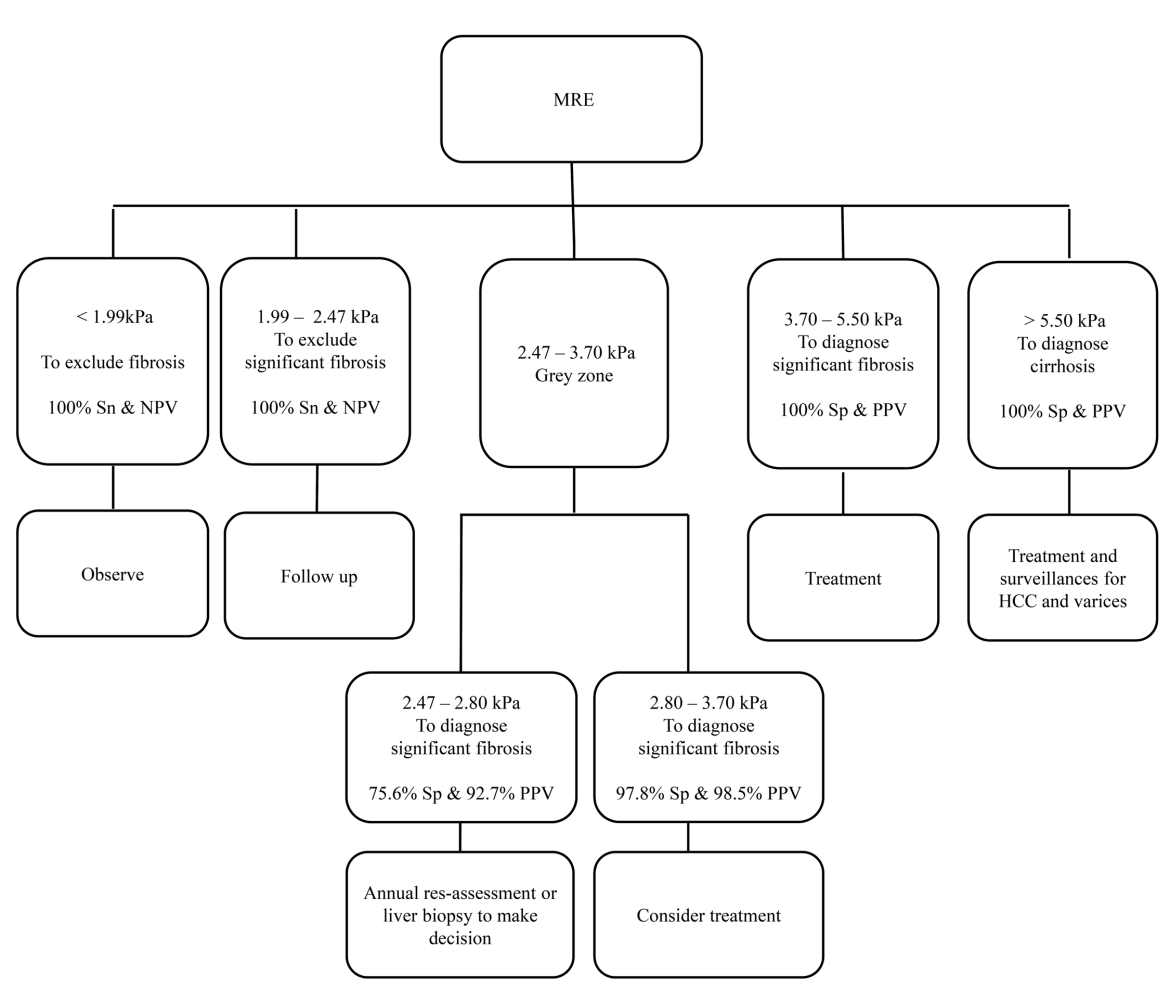
Proposed clinical algorithm of liver stiffness measured by MR Elastography in patients with HBV and those with HCV.

Some previous studies proposed algorithms for ultrasound elastography.[33, 34] According to our results, we proposed a clinical algorithm for the utilization of MRE (Fig 4). We defined different optimal cutoff values of liver stiffness according to different thresholds for sensitivity and specificity. The cutoff value with 100% sensitivity was used to exclude significant liver fibrosis, while the cutoff value with 100% specificity was used to confirm the presence of significant fibrosis or cirrhosis. We found that liver stiffness values < 2.47 kPa indicate no significant liver fibrosis with 100% sensitivity and a negative predictive value for significant fibrosis, meaning these patients do not need treatment and only require observation. For patients with liver stiffness values ranging from 2.47 to 3.70 kPa, antiviral therapy should be considered, particularly if patients have elevated ALT levels or HBV DNA, and for patients with values in the 2.47–2.80 kPa range, annual reassessment or liver biopsy should be considered. For patients with liver stiffness values ranging from 3.70 to 5.50 kPa, antiviral therapy should be considered as stiffness values in that range are indicative of significant fibrosis. In patients with liver stiffness values higher than 5.50 kPa, liver cirrhosis should be diagnosed.

Our study has several limitations. This study was a retrospective study and some of the patients had received percutaneous liver biopsy (74/160). However, liver biopsy is still the "gold standard" in clinical settings. Second, the relatively small number of patients, especially in subgroups of moderate and severe liver steatosis, might limit the ability to investigate the effect of liver steatosis on liver stiffness. Further investigation with a larger number of patients is needed. Third, the fibrosis stages in our study population were not equally distributed. In our study, patients with F3 and F4 comprised more than 50% of the study population. In clinical practice, patients with viral hepatitis usually receive liver biopsy or undergo surgical intervention because of hepatic tumors or advanced liver fibrosis. This tendency might explain the high prevalence of advanced liver fibrosis in our study population.

In conclusion, MRE is an accurate, non-invasive method for estimating hepatic fibrosis stage in patients with viral hepatitis. The etiology of viral hepatitis (HBV vs. HCV) does not appear to affect MRE-measured liver stiffness. Liver steatosis might influence liver stiffness measurements in patients with normal livers or mild liver fibrosis, but has a negligible effect on the cut off value to discriminate normal/mild from significant liver fibrosis. In patients with liver stiffness values ≥ 2.8 kPa, antiviral treatment should be considered.
